# Impact of unintentional coronary angiography on outcomes of emergency surgery in acute type A aortic dissection: a retrospective study

**DOI:** 10.1186/s12872-022-02821-4

**Published:** 2022-08-24

**Authors:** Hao Peng, Wei Liu, Kai-Tao Jian, Yu Xia, Jian-Shi Liu, Li-Zhong Sun, Yun-Qing Mei

**Affiliations:** 1 Department of Cardiovascular and Thoracic Surgery, Tongji Hospital, Tongji University School of Medicine, 389 Xincun Road, Shanghai, 200065 People’s Republic of China; 2 Department of Cardiovascular Surgery, Shanghai DeltaHealth Hospital, 109 Xule Rd, Shanghai, 201702 People’s Republic of China

**Keywords:** Aortic dissection, Coronary angiography, Emergency operation, Antiplatelet therapy, Misdiagnosis

## Abstract

**Background:**

This study investigated the impact of coronary angiography on outcomes of emergency operation in acute type A aortic dissection (ATAAD) patients who were initially misdiagnosed as an acute coronary syndrome.

**Methods:**

From October 2016 to April 2019, 129 patients underwent emergency operation for ATAAD in our institution, including 21 patients (16.3%, coronary angiography group) who received preoperative coronary angiography without knowledge of the ATAAD, and the rest 108 did not (Non-coronary angiography group). Preoperative clinical characteristics, 30-day mortality and postoperative complications were compared. Multivariable logistic regression was performed to confirm the independent prognostic factors for short-term and long-term outcomes.

**Results:**

Patients undergoing coronary angiography had higher prevalence of preoperative hypotension or shock (61.9% vs 35.2%, *P* = 0.022), ischemic changes on electrocardiogram (66.7% vs 37.0%, *P* = 0.012), platelet inhibition (ADP-induced inhibition 92.0% vs 46.0%, *P* = 0.001), and coronary involvement (66.7% vs 30.6%, *P* = 0.002). 30-day mortality was 4.8% versus 9.3% (*P* = 0.84). Coronary angiography group had more intraoperative bleeding (1900 ml vs 1500 ml, *P* = 0.013) and chest-tube drainage on the first postoperative day (1040 ml vs 595 ml, *P* = 0.028). However, preoperative coronary angiography was not independent risk factors for 30-day mortality (OR 0.171, 95%CI 0.013–2.174, *P* = 0.173) and overall survival (HR 0.407; 95%CI 0.080–2.057; *P* = 0.277).

**Conclusion:**

Patients undergoing coronary angiography carried a higher risk of preoperative hemodynamic instability, myocardial ischemia, and perioperative bleeding. However, unintentional coronary angiography did not have a significant impact on short-term and long-term outcomes of emergency surgery in ATAAD.

**Supplementary Information:**

The online version contains supplementary material available at 10.1186/s12872-022-02821-4.

## Background

Acute type A aortic dissection (ATAAD) is one of the most deadly cardiovascular diseases, with a post-onset mortality rate of 1% to 2% increased every hour on the first day without surgical intervention [1]. Emergency surgical repair was proved to be the best therapeutic option in these circumstances [2, 3]. Survival relies on the rapid accurate diagnosis and timely lifesaving emergency operation.

The clinical presentation of ATAAD and acute coronary syndrome (ACS) can be remarkably similar, making diagnosis challenging [4, 5]. Patients with ATAAD were occasionally misdiagnosed as ACS and transferred to a catheterization laboratory to undergo coronary angiography (CA) without knowledge of the ATAAD (unintentional CA) [6–8]. Unintentional CA might result in significant surgical delay, due to the time spent searching of coronary ostia in the diseased aortic root. In addition, the dual antiplatelet administration in preparation for potential coronary intervention significantly increased the risk of coagulopathy and perioperative bleeding, making the decision to operate urgently difficult [9–11]. Previous studies had shown that emergency operation was postponed or even concealed in ATAAD patients who undergoing preoperative CA [12, 13], which undoubtedly increased the risk of death. Furthermore, time consumed by angiography, the instrumentation of the diseased aorta, and the myocardial ischemia by coronary ostium involvement were also associated with increased risk of dissection progression, hemodynamic collapse, and aortic rupture [14–16].

There was little known about the effect of CA on the ATAAD, in most of which the angiography was performed with awareness of ATAAD to clarify concomitant coronary artery disease [12, 14–16]. Because dual antiplatelet medication was not provided and patients with hemodynamic instability were excluded, these deliberate CA had a lower influence on ATAAD patients' prognosis than inadvertent CA. Therefore, the aim of this study was to explore the impact of unintentional CA on the outcomes of emergency surgery in ATAAD patients.

## Methods

### Study population and study design

As the regional aortic center, our tertiary heart hospital (Shanghai DeltaHealth Hospital) received ATAAD patients for emergency surgical intervention from transferring hospitals where the primary diagnosis of aortic dissection was made. The clinical database was queried to include all patients admitted to our institution with a diagnosis of ATAAD and undergoing emergency operations between October 2016 and April 2019.

The exclusion criteria were as follows: (1) the time from onset to admission longer than 7 days; (2) the time from admission to surgery longer than 24 h; (3) emergency operation for giant true aneurysm or type B aortic dissection; (4) patients who were too sick for surgery or refused surgery; (5) patients died before emergency operation. According to whether preoperative coronary angiography was performed, the patients were divided into the CA group and the NCA group. The research protocol for this study was reviewed and approved by the Medical Ethics Committee of Shanghai DeltaHealth Hospital [No. SDH (2020) KYLWZQTYPJ001], and informed consent was approved to be waived for its retrospective nature by the Medical Ethics Committee of Shanghai DeltaHealth Hospital. Medical records were reviewed for data collection. Follow-up was performed by outpatient clinical visit and telephone contact. The last follow-up date was April 30th, 2020.

### Clinical management

**Fig. 1:**
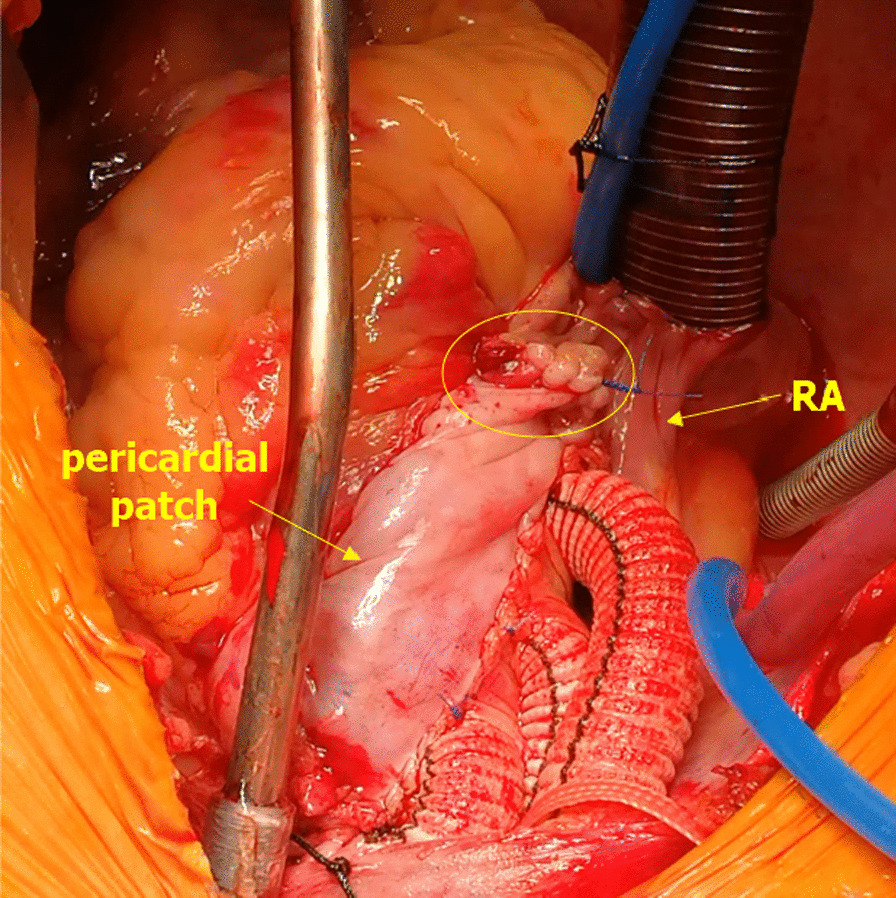
Perigraft to right atrial shunt. The residual autologous aortic wall was wrapped around the prosthetic graft. A pericardial patch was used in case the aortic wall was insufficient. A small opening was then made on the right atrium and anastomosed to the aortic wall and pericardial patch to create a fistula between the perigraft pouch and right atrium (yellow circle)

All procedures were carried out using a median sternotomy incision and on cardiopulmonary bypass (CPB). The hypothermic circulatory arrest was initiated at 25 °C with unilateral selective cerebral perfusion in all patients. The Bentall procedure was performed for proximal aortic repair if there was significant aortic sinus dilation (≥ 4.5 cm) and aortic valve insufficiency. Otherwise, aortic valve repair and ascending aorta replacement were performed. Unless the patient was too weak to tolerate this treatment, total arch replacement and frozen elephant trunk procedures with distal aortic repair were routinely conducted if the aortic arch was involved in the dissection. Partial replacement of the aortic arch was performed to those weak patients. The coronary repair was applied when the coronary ostium was compressed by hematoma in the false lumen. If discontinuity of the ostium or coronary artery occurred, coronary artery bypass grafting (CABG) would be performed. The autologous aortic wall was wrapped around the graft to create a ‘perigraft to right atrial shunt’ following aortic repair in all cases (Fig. 1). Red blood cell was transfused when the blood hemoglobin level decreases to < 70 g/L in stable patients and < 90 g/L in patients with symptomatic anemia. Plasma was transfused in patients with ongoing bleeding and signs of impaired coagulation. Platelet was transfused in patients with ongoing bleeding and suspected platelet dysfunction if available. Re-operation was performed in case of chest tube drainage > 300 ml per hour, a persistent fall in hemoglobin and unstable hemodynamics, or suspected cardiac tamponade.

### Data collection and outcome

Clinical data were collected using a standardized data form and definitions, including patient demographic and medical history, preoperative clinical characteristics, coagulation function, intraoperative data, perioperative bleeding, postoperative complications, and mortality. Intraoperative data included procedures performed for proximal and distal aortic repair and coronary interventions, operative time, and time of cardiopulmonary bypass. The risk of perioperative bleeding was assessed by the volume of intraoperative bleeding, chest tube drainage on the first three postoperative days, blood transfusion, and the rate of re-exploration for bleeding. Postoperative complications included low cardiac output syndrome, cardiac tamponade, new-onset atrial fibrillation, respiratory insufficiency requiring tracheostomy, reintubation, and acute renal failure requiring continuous renal replacement therapy (CRRT), and any neurological deficiency. The length of intensive care unit (ICU) stay and hospital stay, re-admission to ICU, and 30-day re-admission to the hospital were also documented to evaluate the postoperative recovery course. The intramural hematoma was diagnosed in the presence of a circular or crescent-shaped thickening of > 5 mm of the aortic wall in the absence of detectable blood flow in the computed tomography. Coronary involvement was defined as retrograde dissection of the aorta reaching the coronary ostium based on intraoperative findings. Coronary malperfusion was defined as coronary involvement combined with new ST-segment elevation > 0.1 mV, abnormal left ventricular wall motion on echocardiogram, and/or significant elevation of serum tropin I level before surgery.

The outcomes of this study were as follows: (1) 30-day mortality; (2) the risk of perioperative bleeding; (3) postoperative complications rate within 30 days; (4) overall survival (OS).

### Statistical analysis

Statistical analysis was conducted using SPSS statistic 23.0 (SPSS, Chicago, Illinois, USA). Normally distributed continuous variables were expressed as mean ± standard deviation (SD) and analyzed by unpaired Student’s *t*-test. Non-normally distributed continuous data were expressed as median (M) and interquartile range (IQR: Q25–Q75%) and analyzed by Mann–Whitney *U* test. Categorical variables were reported as frequency and analyzed using Chi-square analysis or Fisher's exact test. The Kaplan–Meier method was used to estimate the OS probabilities, and differences were compared using the log-rank test. Univariate analysis was performed to examine the association of each variable with the outcome variable. Binary logistic regression was used to identify independent predictors of 30-day mortality. Survival prognostic factor analysis was performed using the Cox regression model. For both the binary logistic regression model and the Cox regression model, the candidate variables were selected by using stepwise variable selection (*α*_sle_ = 0.05, *α*_sls_ = 0.1). The 'preoperative CA' was then forced to enter the binary logistic regression and the Cox regression model, respectively, with independent predictors of 30-day mortality and overall survival, to identify the impact of preoperative CA on early mortality and long-term survival. Two-tailed *P*-values of < 0.05 were considered significant.

## Results

From October 2016 to April 2019, a total of 321 patients with a diagnosis of aortic dissection were admitted to the Department of Cardiovascular Surgery in Shanghai DeltaHealth Hospital. 295 of these patients underwent the operation, in which 196 patients were diagnosed with type A aortic dissection and the remaining 99 with type B aortic dissection. Within 24 h after admission to the hospital, 129 patients diagnosed with ATAAD underwent operation. One patient had undergone Bentall procedures for ATAAD 8 years ago in the other hospital and suffered from the painful acute onset of dissecting aneurysm of the aortic arch. She underwent a total arch replacement and frozen elephant trunk procedure 37 h following admission and was excluded due to not fulfilling the inclusion criteria.

12 patients with a diagnosis of ATAAD were excluded because they received no surgery, including 7 patients who died suddenly before surgery, 3 patients for their critical state and severe comorbidities (cerebral hemorrhage in 1 patient and mesenteric ischemia in the other 2), and 2 patients due to patient refusal. Among them, three patients (25%) had undergone preoperative CA but died of aortic rupture.

A total of 129 patients were finally included in this study. Preoperative CA was performed on 21 patients (16.3%, CA group) at local hospitals; all of them were initially suspected of having ACS. The diagnosis of aortic dissection was identified during the angiography procedure and confirmed with the following computed tomography. The rest 108 patients (NCA group) were initially diagnosed with aortic dissection by computed tomography without coronary angiography.

### Preoperative clinical characteristics

Baseline demographic and clinical information is presented in Table 1. Patients in the CA group had a male predominance and received significantly more antiplatelet therapy and dual antiplatelet therapy than the NCA group (100% vs 0.9%, *P* < 0.001; 85.7% vs 0%, P < 0.001; respectively). No patients received fibrinolytic treatment in this cohort. Patients in the CA group were significantly more likely to experience preoperative hypotension or shock than those in the NCA group (61.9% vs 35.2%, *P* = 0.022).

**Table 1:** Preoperative clinical characteristics

Variable	CA group (n = 21)	NCA group(n = 108)	P
Male [n (%)]	19 (90.5%)	72 (66.7%)	**0.030**
Age [years, mean ± SD]	54.0 ± 7.8	54.3 ± 13.0	0.865
Body surface area [m^2^, mean ± SD]	1.89 ± 0.20	1.84 ± 0.25	0.332
Cardiac surgery history [n (%)]	0 (0.0%)	5 (4.6%)	0.591
Hypertension [n (%)]	15 (71.4%)	75 (69.4%)	0.856
Diabetes mellitus [n (%)]	1 (4.8%)	3 (2.8%)	0.513
Coronary artery disease history [n (%)]	0 (0%)	2 (1.9%)	1.000
Smoking history [n (%)]	13 (61.9%)	44 (40.7%)	0.074
Intramural hematoma [n (%)]	5 (23.8%)	23 (21.3%)	1.000
Marfan syndrome [n (%)]	0 (0.0%)	10 (15.2%)	0.217
Any antiplatelet therapy [n (%)]	21 (100%)	1 (0.9%)	** < 0.001**
Dual antiplatelet therapy [n (%)]	18 (85.7%)	0 (0.0%)	** < 0.001**
Hypotension/shock [n (%)]	13 (61.9%)	38 (35.2%)	**0.022**
Cardiac tamponade [n (%)]	4 (19.0%)	25 (23.1%)	0.900
Mesenteric malperfusion	1 (4.8%)	3 (2.8%)	0.513
Ischemic changes on ECG [n (%)]	14 (66.7%)	40 (37.0%)	**0.012**
Troponin I [ng/ml, M (IRQ)]	0.048 (0.251)	0.022 (0.161)	0.266
Duration: onset to admission [hours, M (IRQ)]	10.0 (12.0)	11.0 (17.0)	0.297
Duration: admission to operation [hours, M (IRQ)]	1.5 (3.0)	1.5 (2.9)	0.926
Duration: onset to operation [hours, M (IRQ)]	12.0 (17.0)	14.0 (18.8)	0.442

Bold values indicate statistically significant values, *P* < 0.05

An electrocardiogram (ECG) and plasma troponin test were performed on admission. Ischemic changes on preoperative ECG (including ST-segment elevation or depression, T-wave inversion, Q-wave and left bundle branch block) were almost double in the CA group compared to the NCA group (66.7% vs 37.0%, *P* = 0.012). The median plasma level of troponin I in the CA group was numerically higher than in the NCA group but was not statistically significant (0.048 ng/ml vs 0.022 ng/ml, *P* = 0.266).

The other demographics, medical history, and clinical presentations showed no difference between groups. The time from symptom onset to admission (CA 10.0 h vs NCA 11.0 h, *P* = 0.297) and that from admission to operation (CA 1.5 h vs NCA 1.5 h, *P* = 0.926) were similar between the two groups.

## Results of unintentional preoperative coronary angiography

The preoperative CA revealed 11 (52.4%) patients without distinct coronary abnormality, and 6 (28.6%) patients with normal left coronary artery and ambiguous right coronary artery. Further coronary angiography was ceased in the remaining 4 (19.0%) patients due to consideration of aortic dissection after aortography or difficulty in coronary artery identification.

No serious intraoperative complications of CA (including allergic reaction, vascular access site injury, malignant ventricular arrhythmia, iatrogenic coronary or aortic dissection, and cardiac arrest) were documented in all 21 patients.

### Coronary involvement and coronary malperfusion

Coronary involvement was detected in 46 (35.6%) patients in this cohort (14 in the CA group vs 32 in the NCA group), the proportion of which was significantly higher in the CA group (66.7% vs 29.6%, *P* = 0.006). 17 (13.2%) patients were suffered from coronary malperfusion, including 4 in the CA group and 13 in the NCA group. The rate of coronary malperfusion was comparable between the two groups (CA 19.0% vs NCA 12.0%, *P* = 0.606). There was no significant difference in 30-day mortality (11.8% in the malperfusion group vs 8.0% in the non-malperfusion group, *P* = 0.963), overall survival (82.4% in the malperfusion group vs 89.3% in the non-malperfusion group, *P* = 0.404) and postoperative complications between patients with coronary malperfusion or not. Detailed information was attached to Additional file 1: Table S1.

### Preoperative coagulopathy, perioperative bleeding, and blood transfusion

Platelet aggregation inhibition induced by adenosine diphosphate (ADP) and arachidonic acid (AA) was significantly higher in the CA group than in the NCA group (92.0% vs 46.0%, *P* = 0.001; 91.4% vs 71.0%, *P* = 0.042; respectively).

A bolus of 2500U of unfractionated heparin was administered following arterial cannulation during angiography in all patients in the CA group. Heparinized saline was used to flush the sheath and catheter during the CA procedure. However, the activated partial thromboplastin time on admission was similar (CA 30.9 s vs NCA 29.0 s, *P* = 0.327). Other indicators of coagulation were comparable between the two groups (Table 2).

**Table 2:** Preoperative coagulation function, perioperative bleeding, and blood transfusion

Variable	CA group (n = 21)	NCA group(n = 108)	P
*Coagulation function*			
INR [M (IRQ)]	1.04 (0.14)	1.04 (0.15)	0.245
aPTT [seconds, M (IRQ)]	30.9 (7.6)	29.0 (8)	0.327
Fibrinogen [g/L, M (IRQ)]	2.00 (1.25)	2.20 (1.57)	0.318
D-Dimer [mg/L, M (IRQ)]	13.9 (26.8)	9.34 (17.9)	0.697
Platelet count (10^9^/L, mean ± SD)	168.6 ± 62.3	157.5 ± 66.6	0.482
*Thromboelastography*			
R [reaction time, min, M (IRQ)]	5.10 (3.37)	4.70 (2.00)	0.787
K [potentiation phase, min, M (IRQ)]	2.20 (1.10)	1.80 (0.85)	0.305
α [rate of clot, degree, M (IRQ)]	61.0 (21.7)	62.4 (17.5)	0.411
MA [maximum amplitude, mm, M (IRQ)]	60.0 (12.0)	61.3 (11.5)	0.679
LY30 [fibrinolysis, %, M (IRQ)]	0.00 (0.10)	0.00 (0.05)	0.822
AA-induced platelet inhibition [%, M (IRQ)]	92.0 (43.6)	46.0 (44.2)	**0.001**
ADP-induced platelet inhibition [%, M (IRQ)]	91.4 (36.4)	71.0 (55.4)	**0.042**
*Perioperative bleeding*			
Intraoperative bleeding [ml, M (IRQ)]	1900 [1925]	1500 [1100]	**0.013**
*Chest tube drainage [ml, M (IRQ)]*			
Operative day	1040 [1030]	595 [685]	**0.028**
1st postoperative day	460 [477.5]	320 [440] ^&^	0.567
2nd postoperative day	150 [280]	130 [204] ^#^	0.642
Re-exploration for bleeding [n (%)]	3 (14.3%)	14 (13.0%)	1.000
*Blood transfusion*			
RBC [U, M (IRQ)]	8 (14)	6 (10)	0.149
Plasma [U, M (IRQ)]	18 (25)	12 (14)	0.075
Platelets [U, M (IRQ)]	1 (2)	0 (1)	**0.003**

Bold values indicate statistically significant values, *P* < 0.05

INR, international normalized ratio; aPTT, activated partial thromboplastin time; ADP, adenosine diphosphate; AA, arachidonic acid; RBC, red blood cell. ^&^Missing data due to death of patients, n = 107. ^#^ Missing data due to death of patients, n = 106

Median intraoperative bleeding volumes in the CA group were significantly higher than in the NCA group (1900 ml vs 1500 ml, *P* = 0.013). Volumes of chest tube drainage were also significantly higher than the NCA group on the operative day (1040 ml vs 595 ml, *P* = 0.028). However, chest tube drainage was comparable on the first and second postoperative days between groups. There was no significant difference in re-operation for bleeding and postoperative cardiac tamponade between the two groups.

Volumes of platelet transfusion in the CA group were significantly more than NCA group (1U vs 0U, *P* = 0.003) whereas no difference was found in transfusion of red blood cell (RBC) (CA 8U vs NCA 6U, *P* = 0.149) and plasma (CA 18U vs NCA 12U, *P* = 0.075).

### Intraoperative interventions

No differences were identified between the two groups in the proximal or distal aortic repair strategies (Table 3). The CA group had significantly more coronary repairs than the NCA group (61.9% versus 25.9%, *P* = 0.001), but CABG was comparable between the groups (CA 4.8% vs NCA 3.7%, *P* = 1.000). The operative time and time of cardiopulmonary bypass were similar among the groups.

**Table 3:** Surgical intervention

Variable	CA group (n = 21)	NCA group (n = 108)	P
*Proximal aortic repair*			
Bentall procedure [n (%)]	4 (19.0%)	34 (31.5%)	0.253
Aortic valvuloplasty [n (%)]	7 (33.3%)	25 (23.1%)	0.323
Ascending aorta replacement [n (%)]	17 (81%)	70 (64.8%)	0.205
*Distal aortic repair*			
Hemi-arch replacement [n (%)]	1 (4.8%)	16 (14.8%)	0.372
Sun’s procedure [n (%)]	18 (85.7%)	73 (67.6%)	0.096
*Coronary intervention*			
Ostium repair [n (%)]	13 (61.9%)	28 (25.9%)	**0.001**
CABG [n (%)]	1 (4.8%)	4 (3.7%)	1.000
Overall intervention [n (%)]	14 (66.7%)	32 (29.6%)	**0.006**
Operative time [minutes, M (IRQ)]	319 (187.5)	323.5 (112.3)	0.674
Time of CPB [minutes, M (IRQ)]	168.0 (47.5)	160.5 (47.6)	0.794
Time of ACC [minutes, M (IRQ)]	86.0 (30.0)	87.5 (24.0)	0.728
Time of DHCA [minutes, M (IRQ)]	17.0 (6.0)	18.0 (7.8)	0.627

Bold values indicate statistically significant values, *P* < 0.05

CABG, coronary artery bypass grafting; CPB, cardiopulmonary bypass; ACC, aortic crossclamping; DHCA, deep hypothermic circulatory arrest; RBC: red blood cell; Sun’s procedure: total arch replacement + frozen elephant trunk procedure

### Survival outcomes and postoperative complications

**Fig. 2:**
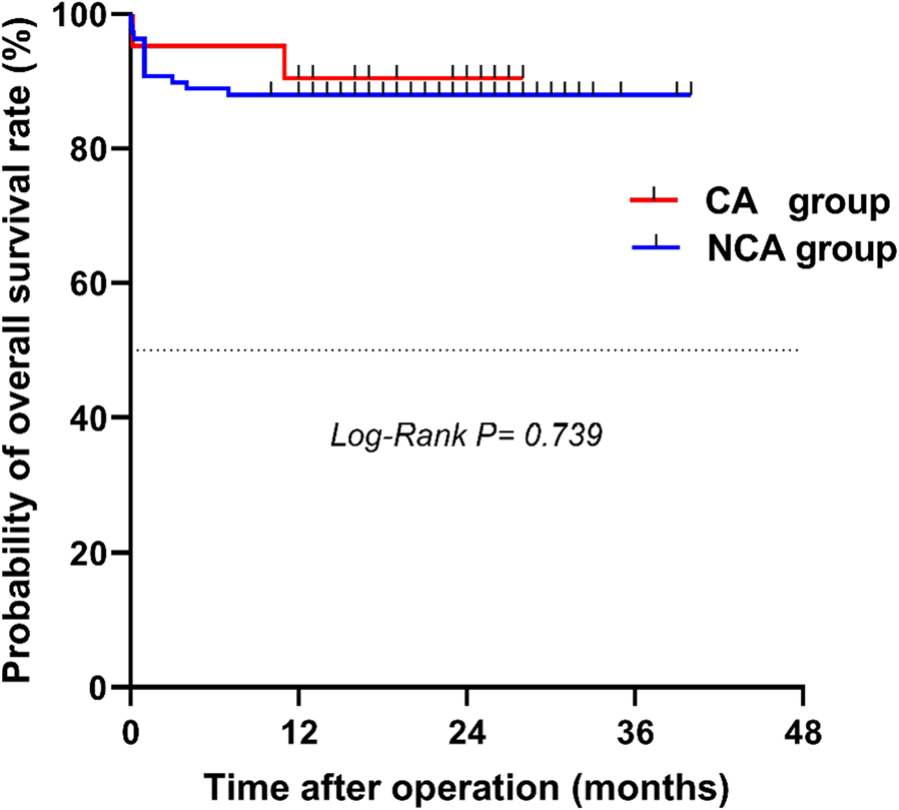
Survival analysis showed no significant difference in overall survival between the two groups

30-day mortality was not significantly different between the CA and NCA groups (4.8% vs 9.3%, *P* = 0.804). The follow-up time was 19 [13] months. Two patients were lost to follow-up after 30 days post discharge, with a follow-up rate of 98.4%. No significant differences in accumulative overall survival were found between the CA and NCA groups (90.5% vs 88.0%, *P* = 0.739) (Fig. 2).

Overall, there were no significant differences in major postoperative complications. However, the incidence of ICU readmission (CA 30.0% vs NCA 7.7%, *P* = 0.012) and new-onset atrial fibrillation (CA 47.6% vs NCA 19.4%, *P* = 0.006) were significantly higher in the CA group than in the NCA group (Table 4). The median length of hospital stay was numerically much longer in the CA group than the NCA group, although did not reach statistical significance (18.0 days vs 13.0 days, *P* = 0.056). The echocardiography performed before discharge revealed no differences in the left ventricular ejection fraction, the fractional shortening, and the incidence of wall motion abnormalities between groups. The median brain natriuretic peptide (BNP) before discharge was also comparable across the groups (CA 329.0 pg/ml vs NCA 297.0 pg/ml, *P* = 0.443).

**Table 4:** Surgical mortality and postoperative complications

Variable	CA group (n = 21)	NCA group (n = 108)	P
Surgical mortality [n (%)]	1 (4.8%)	10 (9.3%)	0.804
*Cardiac complications*			
Low cardiac output syndrome [n (%)]	0 (0.0%)	6 (5.6%)	0.588
Cardiac tamponade [n (%)]	2 (9.5%)	13 (12.0%)	1.000
New-onset atrial fibrillation [n (%)]	10 (47.6%)	21 (19.4%)	**0.006**
*Echocardiogram before discharge*			
LV ejection fraction [%, mean ± SD]	60.9 ± 6.9	61.3 ± 7.8	0.834
LV fractional shortening [%, mean ± SD]	32.7 ± 4.8	32.9 ± 5.5	0.860
LV Wall motion abnormality [n (%)]	2 (9.5%)	13(12.1%)	1.000
Re-intubation [n (%)]	2 (9.5%)	13 (12.0%)	1.000
Tracheotomy [n (%)]	2(9.5%)	12(11.1%)	1.000
Acute renal failure [n (%)]	2 (9.5%)	6 (5.6%)	0.845
Coma [n (%)]	1 (4.8%)	10 (9.3%)	0.804
Hypoxic-ischemic encephalopathy [n (%)]	1 (4.8%)	6 (5.6%)	1.000
Cerebral infarction [n (%)]	2 (9.5%)	13(12.0%)	1.000
Cerebral hemorrhage [n (%)]	1(4.8%)	3(2.8%)	0.513
ICU stay [hours, M (IRQ)]	92.0 (129.8)	63.5 (68,7)	0.193
ICU readmission* [n (%)]	6 (30.0%)	8 (7.7%)	**0.012**
Hospital stay [days, M (IRQ)]	18.0 (16.5)	13.0 (10.0)	0.056
30-day readmission* [n (%)]	2 (10%)	17 (16.3%)	0.702

Bold values indicate statistically significant values, *P* < 0.05

ICU, intensive care unit. *As patients died in ICU were excluded, 20 in the CA group and 104 patients in NCA group were included

### Multivariate analysis

Multivariate analysis showed that intramural hematoma (OR 7.375; 95% CI 1.048–51.884, *P* = 0.045), CPB time (OR 1.023; 95% CI 1.005–1.042; *P* = 0.012), postoperative hemodialysis (OR 59.610; 95% CI 7.748–458.613; P < 0.001), and cerebral hemorrhage (OR 63.181; 95% CI 4.067–981.416; P <  = 0.003) were independent risk factors for 30-day mortality. Coronary angiography was not a significant risk factor for 30-day mortality after adjusting for the above variables (OR 0.171, 95%CI 0.013–2.174, *P* = 0.173).

Cox regression model showed that preoperative creatinine (HR 1.006; 95%CI 1.003–1.009; P < 0.001), preoperative lactic acid (HR 1.487; 95%CI 1.260–1.756; P < 0.001), intramural hematoma (HR 11.767; 95%CI 2.707–51.143; *P* = 0.001), CPB time (HR 1.017; 95%CI 1.007–1.027; *P* = 0.001), reintubation (HR 5.262; 95%CI 1.028–26.936; *P* = 0.046), and postoperative cerebral hemorrhage (HR 27.164; 95%CI 3.312–222.826; *P* = 0.002) were independent prognostic factors for OS. Preoperative coronary angiography was not a significant prognostic factor for OS after adjusting for the above variables (HR 0.407; 95%CI 0.080–2.057; *P* = 0.277).

## Discussion

Angiography had once been considered useful for confirming the diagnosis and defining coronary anatomy in patients with aortic dissection [17, 18]. Given that delayed surgery and instrumentation of the diseased aorta increase the risk of dissection progression, cardiac tamponade, and aortic rupture, it is no longer generally advised for ATAAD patients in the age of noninvasive imaging [14–16]. Nonetheless, because the clinical presentation of the two diseases is so similar, individuals with ATAAD may be sent to the catheterization laboratory due to a misdiagnosis of ACS. The rate of preoperative coronary angiography employment for patients with ATAAD was observed to be 9.3% to 21.9% [6, 12, 19]. However, the diagnosis of ATAAD had either already been confirmed or not well documented when the coronary angiographies were performed in these studies. Thus, the present study focused on the impact of coronary angiography performed without knowledge of the ATAAD on outcomes of emergency surgery in ATAAD. Based on our results, 16.3% of ATAAD patients underwent coronary angiography due to misdiagnosis of ACS, indicating a relatively common scenario in this population.

The CA group exhibited considerably greater ischemic changes on preoperative ECG and more coronary involvement, indicating that patients undergoing unintentional coronary angiography were at a higher risk of myocardial ischemia. Preoperative hypotension or shock was also more common in the CA group, reflecting the hemodynamic compromise in these patients. As the incidence of cardiac tamponade was comparable across groups, myocardial ischemia might be the dominant cause of hemodynamic changes. Interestingly, the value of troponin did not achieve a significant difference between groups. These findings were consistent with the results of Pourafkari, who found no difference in troponin increase in ATAAD patients with acute ECG changes [19]. The possible mechanism of this phenomenon might lie in that compromise of the coronary perfusion would be intermittent, incomplete, and variable when it was caused by coronary ostium obstruction of the local dissection flap instead of complete coronary detachment [18, 20]. This hypothesis was also supported by our comparable incidence of CABG applied between the two groups, which was performed only in case of complete avulsion of the coronary artery [18, 21].

Patients receiving accidental coronary angiography were exposed to an increased risk of perioperative coagulopathy and bleeding due to the administration of loading dose of antiplatelet medications before to angiography, which led mostly to the emergency operation delay or cancellation [22]. The higher inhibition of platelet aggregation and the more volume of intraoperative bleeding and chest tube drainage on the operative day demonstrated a higher risk of bleeding in the CA group in the early perioperative period. These findings were consistent with other studies showing the association of bleeding and antiplatelet therapy [23, 24]. However, there was no significant difference between the two groups to the re-exploration for bleeding or the total amount of RBCs and plasma transfusion, suggesting the difference in bleeding had not resulted in severe clinical consequences. This might likely be attributed to our surgical strategy during emergency aortic operations. When vascular reconstruction had been done, the aneurysmal wall was routinely wrapped around the graft and a fistula was made on the right atrium to drain oozing blood from the aneurysmal sac, which significantly reduced the volume of chest drainage and the subsequent requirement of transfusion. In this way, we achieved good control of bleeding even in patients with antiplatelet therapy and a shortage of platelets in case of emergency.

Our results did not show significant time delay from onset to operation in the CA group, which differed from some published studies. It was reported that delays in correct diagnosis were quite common in patients with initial misdiagnosis of ATAAD [25, 26], and delay to surgery was popular and associated with the poor prognosis in ATAAD patients undergoing intentional preoperative coronary angiography [15, 16, 27]. These differences could be explained in part by the strict time requirement for coronary angiography based on the current guideline for ACS [28], thus minimizing time consumption in the emergency room on detection of the definitive diagnosis. Furthermore, unintentional coronary angiographies were always be ceased immediately upon suspicion of ATAAD, which meant a much shorter time in the catheterization laboratory as compared to intentional coronary angiography. According to our institutional protocols, all the involved patients were transferred to the operating theater as soon as possible no matter the antiplatelet therapy was administered or not (median time from admission to the operation were 1.5 h in both groups), which avoided delayed treatment due to the surgical strategies.

Although unintentional coronary angiography was associated with the risk of hemodynamic instability, myocardial ischemia, and perioperative bleeding, the increase in 30-day mortality was not significant, which proved the necessity and effectiveness of emergent surgical management in this scenario. The surgical delay was considered the main cause of poor hospital mortality in patients who underwent the coronary angiography performed with knowledge of the ATAAD diagnosis, which was not the case in the present study [16, 27]. Antiplatelet therapy was also reported to be associated with increased early mortality in ATAAD patients with initial misdiagnosis, attributed to the increased perioperative bleeding and transfusion [23, 25]. Although the early perioperative bleeding was greater in the CA group, the transfusion of RBC and plasma were comparable between the groups, suggesting that perioperative bleeding in this study appears to have a relatively minor impact on clinical outcomes.

In our study, unintentional coronary angiography was associated with higher rates of ICU readmission (30.0% vs 7.7%, *P* = 0.012) and a trend toward the longer length of ICU stay (92.0 h vs 63.5 h, *P* = 0.193) and hospital stay (18 days vs 13 days, *P* = 0.056), reflecting a more arduous course of recovery. Previous studies had described that preoperative hypotension or shock and the major bleeding within the initial 24 h were independent prognostic factors of in-hospital mortality, while no significant abnormal ECG findings predicted excellent in-hospital survival [15, 29]. Considering that patients undergoing coronary angiography had been exposed to such risk factors, the slower recovery process was easy to understand, and the value of timely and appropriate operative interventions in this circumstance was confirmed.

We did not find the long-term effect of unintentional coronary angiography on prognosis. Ramanath and colleagues (2011) reported increased trends in long-term mortality among patients undergoing preoperative coronary angiography and speculated more preexisting coronary artery disease in this population as the potential underlying cause [12]. Patients in the present study underwent coronary angiography due to misdiagnosis instead of clarifying the coronary anatomy, thus they were younger and had fewer comorbidities such as diabetes mellitus and coronary artery disease. It was reasonable that they had similar long-term survival compared with patients without coronary angiography after the early postoperative phrase of critical illnesses.

In conclusion, unintentional coronary angiography was not uncommon in patients with ATAAD and was associated with a higher risk of perioperative bleeding, myocardial ischemia, and hemodynamic instability. However, the 30-day mortality and long-term survival were not significantly influenced by unintentional coronary angiography given timely and appropriate surgical repairs.

## Limitations

The limitations of this study are its retrospective nature and a relatively small number of subjects. In addition, all patients were initially presented to their local hospitals and transferred to our center for further surgical treatment. There could be inherent selection bias, as critically ill patients could have no opportunity to be transferred or die before admission to our hospital. As mortality was proved better in high-volume centers compared to low-volume centers, and patients transferred to high-volume centers had similar outcomes as direct admits, transferring patients to the center of ‘aortic teams’ was common and recommended nowadays [3, 30]. Our results reflected the prognosis of ATAAD patients in this ‘real world’ scenario.

## Supplementary Information

**Additional file 1: Table S1.** 30-day mortality and postoperative complications in patients with or without coronary malperfusion. **Table S2.** 30-day mortality and postoperative complications in patients with or without coronary artery bypass grafting. **Table S3.** Factors included in the univariate analysis in the Cox regression model.

## Data Availability

All data generated or analyzed during this study are included in this published article.
