# Supplementary material for: Impact of unintentional coronary angiography on outcomes of emergency surgery in acute type A aortic dissection: a retrospective study
Source: BMC Cardiovasc Disord. 2022 Aug 24;22:383. doi: 10.1186/s12872-022-02821-4 (PMC9400216; doi:10.1186/s12872-022-02821-4)
Supplement: Supplementary file 1 — Additional file 1: Table S1. 30-day mortality and postoperative complications in patients with or without coronary malperfusion. Table S2. 30-day mortality and postoperative complications in patients with or without coronary artery bypass grafting. Table S3. Factors included in the univariate analysis in the Cox regression model. [file 12872_2022_2821_MOESM1_ESM.docx]

TABLE S1. 30-day mortality and postoperative complications in patients with or without coronary malperfusion.

| **Variable** | **MP group (n=17)** | **NMP group (n=112)** | **P** |
| --- | --- | --- | --- |
| 30-day mortality [n (%)] | 2 (11.8%) | 9 (8.0%) | 0.963 |
| Overall survival (%) | 82.4% | 89.3% | 0.404 |
| Cardiac complications |  |  |  |
| Low cardiac output syndrome [n (%)] | 21 (5.9%) | 5 (4.5%) | 0.579 |
| Cardiac tamponade [n (%)] | 2 (11.8%) | 13 (11.6%) | 1.000 |
| New-onset atrial fibrillation [n (%)] | 7 (41.2%) | 24 (21.4%) | 0.141 |
| Echocardiogram before discharge |  |  |  |
| LV ejection fraction [%, mean±SD] | 58.6±6.5 | 61.6±7.7 | 0.145 |
| LV Wall motion abnormality [n (%)] | 3 (17.6%) | 12 (10.7%) | 0.671 |
| Re-intubation [n (%)] | 3 (17.6%) | 12 (10.7%) | 0.671 |
| Tracheotomy [n (%)] | 2 (11.8%) | 12 (10.7%) | 1.000 |
| Acute renal failure [n (%)] | 1 (5.9%) | 7 (6.3%) | 1.000 |
| Coma [n (%)] | 1 (5.9%) | 10 (8.9%) | 1.000 |
| Hypoxic-ischemic encephalopathy [n (%)] | 1 (5.9%) | 6 (5.4%) | 1.000 |
| Cerebral infarction [n (%)] | 2 (11.8%) | 13 (11.6%） | 1.000 |
| Cerebral hemorrhage [n (%)] | 1 (5.9%) | 3 (2.7%) | 0.436 |

MP, malperfusion; NMP, non-malperfusion; LV, left ventricle; ICU, intensive care unit.

TABLE S2. 30-day mortality and postoperative complications in patients with or without coronary artery bypass grafting

| **Variable** | **CABG group (n=5)** | **NCABG group (n=124)** | **P** |
| --- | --- | --- | --- |
| 30-day mortality [n (%)] | 0 (0.0%) | 11 (8.9%) | 1.000 |
| Overall survival (%) | 80.0% | 88.7% | 0.608 |
| Cardiac complications |  |  |  |
| Low cardiac output syndrome [n (%)] | 0 (0.0%) | 6 (4.8%) | 1.000 |
| Cardiac tamponade [n (%)] | 1 (20.0%) | 14 (11.3%) | 0.467 |
| New-onset atrial fibrillation [n (%)] | 2 (40.0%) | 29 (23.4%) | 0.750 |
| Echocardiogram before discharge |  |  |  |
| LV ejection fraction [%, mean±SD] | 58.6±10.6 | 61.3±7.51 | 0.434 |
| LV Wall motion abnormality [n (%)] | 2 (40.0%) | 13 (10.5%) | 0.103 |
| Re-intubation [n (%)] | 1 (20.0%) | 14 (11.3%) | 0.467 |
| Tracheotomy [n (%)] | 1 (20.0%) | 13 (10.5%) | 0.442 |
| Acute renal failure [n (%)] | 0 (0.0%) | 8 (6.5%) | 1.000 |
| Coma [n (%)] | 0 (0.0%) | 11 (8.9%) | 1.000 |
| Hypoxic-ischemic encephalopathy [n (%)] | 1 (20.0%) | 6 (4.8%) | 0.247 |
| Cerebral infarction [n (%)] | 2 (40.0%) | 13 (10.5%） | 0.103 |
| Cerebral hemorrhage [n (%)] | 0 (0.0%) | 4 (3.2%) | 1.000 |

CABG, coronary artery bypass grafting; NCABG, non- coronary artery bypass grafting; LV, left ventricle; ICU, intensive care unit.

TABLE S3. Factors included in the univariate analysis in the Cox regression model.

| **Parameters** | **Hazard Ratio** | **95%CI** | **P Value** |
| --- | --- | --- | --- |
| Preoperative paraplegia | 7.326 | 0.958-56.032 | 0.055 |
| Preoperative white blood cell | 1.142 | 1.018-1.280 | 0.023 |
| Preoperative alanine aminotransferase | 1.001 | 1.000-1.002 | 0.017 |
| Preoperative creatinine | 1.005 | 1.003-1.007 | <0.001 |
| Preoperative international normalized ratio | 5.861 | 0.932-36.846 | 0.059 |
| Preoperative lactic acid | 1.225 | 1.119-1.341 | <0.001 |
| Intramural hematoma | 3.380 | 1.225-9.326 | 0.019 |
| Cardiopulmonary bypass time | 1.019 | 1.010-1.028 | <0.001 |
| Aortic crossclamping time | 1.025 | 1.007-1.043 | 0.007 |
| Operative time | 1.007 | 1.003-1.011 | 0.002 |
| Volume of red blood cell transfusion | 1.090 | 1.043-1.139 | <0.001 |
| Volume of plasma transfusion | 1.014 | 1.001-1.027 | 0.029 |
| Volume of platelet transfusion | 2.174 | 1.369-3.453 | 0.001 |
| ICU stay | 1.003 | 1.001-1.004 | <0.001 |
| Re-intubation | 6.457 | 2.333-17.872 | <0.001 |
| Tracheotomy | 3.073 | 0.977-9.665 | 0.055 |
| Postoperative acute renal failure | 9.580 | 3.247-28.267 | <0.001 |
| Postoperative cerebral hemorrhage | 9.013 | 2.518-32.262 | 0.001 |
| Postoperative coma | 6.522 | 2.221-19.155 | 0.001 |
